# Effects of maternal diet-induced obesity on metabolic disorders and age-associated miRNA expression in the liver of male mouse offspring

**DOI:** 10.1038/s41366-021-00985-1

**Published:** 2021-10-18

**Authors:** Laís Vales Mennitti, Asha A. M. Carpenter, Elena Loche, Lucas C. Pantaleão, Denise S. Fernandez-Twinn, Josca M. Schoonejans, Heather L. Blackmore, Thomas J. Ashmore, Luciana Pellegrini Pisani, John A. Tadross, Iain Hargreaves, Susan E. Ozanne

**Affiliations:** 1 Department of Bioscience, Laboratory of Nutrition and Endocrine Physiology, Federal University of São Paulo, Santos, 11015-020 Brazil; 2 Metabolic Research Laboratories and MRC Metabolic Diseases Unit, University of Cambridge, Cambridge, CB2 OQQ United Kingdom; 3 Department of Pathology, University of Cambridge, Cambridge, CB2 1QP United Kingdom; 4 Department of Pharmacy and Biomolecular Science, Liverpool John Moores University, Byrom Street, Liverpool, L3 5UA United Kingdom

**Keywords:** Animal disease models, Fats

## Abstract

**Objective:**

This study investigated the effect of maternal obesity on aged-male offspring liver phenotype and hepatic expression of a programmed miRNA.

**Methods:**

A mouse model (C57BL/6 J) of maternal diet-induced obesity was used to investigate fasting-serum metabolites, hepatic lipid content, steatosis, and relative mRNA levels (RT-PCR) and protein expression (Western blotting) of key components involved in hepatic and mitochondrial metabolism in 12-month-old offspring. We also measured hepatic lipid peroxidation, mitochondrial content, fibrosis stage, and apoptosis in the offspring. To investigate potential mechanisms leading to the observed phenotype, we also measured the expression of miR-582 (a miRNA previously implicated in liver cirrhosis) in 8-week-old and 12-month-old offspring.

**Results:**

Body weight and composition was similar between 8-week-old offspring, however, 12-month-old offspring from obese mothers had increased body weight and fat mass (19.5 ± 0.8 g versus 10.4 ± 0.9 g, *p* < 0.001), as well as elevated serum levels of LDL and leptin and hepatic lipid content (21.4 ± 2.1 g versus 12.9 ± 1.8 g, *p* < 0.01). This was accompanied by steatosis, increased Bax/Bcl-2 ratio, and overexpression of p-SAPK/JNK, *Tgfβ1, Map3k14*, and *Col1a1* in the liver. Decreased levels of Bcl-2, p-AMPKα, total AMPKα and mitochondrial complexes were also observed. Maternal obesity was associated with increased hepatic miR-582-3p (*p* < 0.001) and miR-582-5p (*p* < 0.05). Age was also associated with an increase in both miR-582-3p and miR-582-5p, however, this was more pronounced in the offspring of obese dams, such that differences were greater in 12-month-old animals (−3p: 7.34 ± 1.35 versus 1.39 ± 0.50, *p* < 0.0001 and −5p: 4.66 ± 1.16 versus 1.63 ± 0.65, *p* < 0.05).

**Conclusion:**

Our findings demonstrate that maternal diet-induced obesity has detrimental effects on offspring body composition as well as hepatic phenotype that may be indicative of accelerated-ageing phenotype. These whole-body and cellular phenotypes were associated with age-dependent changes in expression of miRNA-582 that might contribute mechanistically to the development of metabolic disorders in the older progeny.

## Introduction

Global incidence of obesity has increased dramatically in all age groups, including women of reproductive age [1, 2]. A 2019 report in the United Kingdom revealed that more than half of pregnant women were overweight or obese [3]. Maternal obesity during pregnancy has been shown to have long-term “programmed” effects on the offspring [4, 5]. This involves changes in gene expression and permanent structural and functional changes in tissues that make the offspring susceptible to obesity and related diseases [6–8]. These effects of maternal obesity could be mediated by the maternal nutritional status and/or dietary composition [5, 9–11].

Epigenetic processes are thought to be major determinants of programming mechanisms. The major epigenetic determinants are DNA methylation, histone modification and noncoding RNAs, such as microRNAs (miRNAs) [5]. MiRNAs are small molecules that act to repress messenger RNA (mRNA) translation by binding to complementary sequences in the 3′-untranslated region (UTRs) of target mRNAs [12, 13]. It is predicted that at least 30% of all human genes are regulated by miRNAs and disturbances in miRNA expression have been linked to several metabolic conditions, including type-2 diabetes, cardiovascular disease (CVD), lipid-homeostasis impairment, and liver steatosis and fibrosis [14–16]. For example, increased hepatic miR-582 expression has been reported in response to high-fructose diet consumption [17] and cirrhotic livers [18]. Growing evidence indicates that maternal obesity and/or high-fat feeding may program the expression of different miRNAs in various tissues, directly affecting the health status of the offspring [19–23]. However, these studies have generally focussed on a single time point in young-adult life.

The aging process is thought to start before birth and has been associated with development of programmed phenotypes [24]. Individuals exposed to a suboptimal maternal environment (e.g., maternal obesity) during early critical periods are more susceptible to age-associated diseases in later life, such as obesity, type-2 diabetes, and CVD [24]. Rodríguez-González et al. revealed that progeny exposed to maternal obesity were predisposed to premature aging and the progression of nonalcoholic fatty liver disease (NAFLD) in a sex-specific manner [25]. However, most published animal studies on the effects of maternal obesity and/or excessive consumption of high-fat/high-sugar diets during pregnancy and lactation focused on young-adult offspring with a limited number focussing on the reproductive system and cerebrovasculature of aged offspring [26–28]. Therefore, this study aimed to investigate the liver phenotype of mice at 12 months of age born to obese dams and to explore if this was related to changes in the miRNA expression with advancing of age.

## Materials and methods

### Animals and diet

All animal experiments were conducted at the University of Cambridge according to the UK Home Office Animals (Scientific Procedures) Act 1986 Amendment Regulations 2012, following ethical review and approval by the University of Cambridge Animal Welfare and Ethical Review Board. This study is based on an established model of maternal diet-induced obesity [29] where female C57BL/6 mice are fed either a standard laboratory chow (RM1, control diet) [~7% simple sugars, 3% fat, 50% polysaccharide, and 15% protein (wt/wt)] or an obesogenic diet [high-fat diet: ~10% simple sugars, 20% animal lard, 28% polysaccharide, and 23% protein (wt/wt) supplemented with sweetened condensed milk (~55% simple sugar, 8% fat, and 8% protein (wt/wt)) and micronutrient mineral mix] from weaning (both diets from SDS Diets, UK). Proven breeders [29] were mated when controls had a total body-fat mass less than 5 g, and the obese exceeded 10 g of total fat mass as assessed by time domain nuclear magnetic resonance (TD-NMR) (Mini-spec TD-NMR, Bruker UK Ltd). The presence of a copulatory plug indicated day 1 of pregnancy. Females remained on their respective diet throughout pregnancy and lactation under standard conditions of 12-h light and 12-h dark at 23 °C. Assignment of dietary groups was performed by a technician not involved in the molecular analysis. To control the nutritional plane, litter size was standardized on postnatal day 2 to six pups. On day 21, male offspring were weaned onto and maintained on control diet. For all outcome measurements only one animal per litter was included in the analysis, therefore, *n* refers both to the number of offspring included and the number of litters represented. Offspring groups are referred to as CC (offspring born to control dams) and OC (offspring born to obese dams). Body weight of male offspring was assessed from three weeks, whereas body composition by TD-NMR was measured from four weeks of age. Both were recorded weekly up to 12 weeks of age and monthly up to 52 weeks of age.

To investigate the contribution of advancing age on the expression of miR-582 (−3p and −5p), two different time points (eight weeks and 12 months of age) were included for this measurement. Mice at eight weeks of age are considered young adults (sexual maturity is attained by approximately six weeks of age), and at 12 months of age, they are deemed older adults (middle age). The average life span of C57/Bl6 laboratory mice is about 24 months of age [30]. At 12 months of age, blood glucose was measured using a glucometer (AlphaTRAK II, Abbott Logistics BV, NL). Animals were killed by rising CO_2_ concentration. Various tissues were collected at eight weeks of age (*n* = 8 for each experimental group); however, only livers and body composition were analyzed for this study. For 12-month-old offspring (*n* = 9 for each experimental group), blood was collected by cardiac puncture for serum-metabolite analysis. Adipose-tissue depots (intra-abdominal, retroperitoneal, and epididymal) and liver were dissected, weighed, snap-frozen, and stored at −80 °C until use.

### Serum analysis

Insulin and leptin concentrations were measured using enzyme-linked immunosorbent assay (ELISA) (Crystal Chem, USA). Total cholesterol and triglycerides (TG) were measured using a Dimension RxL analyzer (Siemens Healthcare Limited, UK). HDL cholesterol was measured using a homogeneous accelerator-selective detergent assay with a Dimension RxL analyzer (Siemens Healthcare Limited, UK). LDL cholesterol was calculated using the Friedwald equation [31]. Free fatty acids were measured using the Roche Free Fatty Acid Half-Micro Kit (Roche Diagnostics Limited, UK). Homeostatic Model Assessment for Insulin Resistance (HOMA-IR) was calculated using the formula: HOMA-IR = [fasting insulin (mU L^−1^) × fasting glucose (mmol L^−1^)]/22.5.

### Glucose-tolerance test (GTT)

At 12 months of age, offspring were fasted (16 h) and tail blood glucose measured (AlphaTRAK II, Abbott Logistics BV, NL). Animals were injected intraperitoneally with a 10% (wt/vol.) glucose solution (1 g/kg body weight) and tail blood glucose measured 15, 30, 60, 120, and 180 min after injection. Area under the curve (AUC) was calculated using the trapezoids rule (GraphPad Prism 7.02 for Windows, GraphPad Software, USA).

### Quantification of liver lipid content and histological analysis

Accumulation of hepatic lipid was determined using a modified Folch method [32]. For histological analysis, liver tissue was fixed in 10% neutral-buffered formalin for 48 hours before processing and paraffin embedding. For evaluation of hepatic steatosis, sections (7 µm) were stained with hematoxylin and eosin (H&E) and images were captured using a ZEISS Axio Scan.Z1 slide scanner. Steatosis was graded by a histopathologist blinded to the offspring group, and scored based on the percentage of hepatocytes showing steatosis (macro- or microvesicular) into four different categories: absent = <5% (grade 0), mild = <30% (grade 1), moderate = <60% (grade 2), and severe = >60% (grade 3) [33].

For quantification of collagen deposition, sections (5 µm) were stained with Picrosirius Red and images were captured using a ZEISS Axio Scan.Z1 slide scanner. Collagen staining was calculated by converting images to an L*a*b stack and analyzing red stain on dimension “a” using ImageJ software (version 1.52 g, National Institute of Health, USA). Five fields per section were analyzed and the average collagen staining recorded. These sections were also used to fibrosis-stage assessment (by a histopathologist blinded to the offspring group) according to the classification described by Kleiner et al. [34] as follows: absent = no increase in fibrosis, mild = centrilobular/pericellular fibrosis only, moderate = centrilobular + portal fibrosis, and severe = bridging fibrosis.

### TUNEL assay

Liver apoptosis was assessed by TUNEL assay using paraffin-embedded sections. An in situ apoptosis detection kit (#MK500, Takara Bio Inc., Japan) was used to detect DNA fragments generated by apoptosis as per the manufacturer’s instructions. Images were captured using a ZEISS Axio Scan.Z1 slide scanner. TUNEL-positive cells were detected based on the average DAB (brown) staining within each cell nuclei using an embedded algorithm in QuPath v0.2.3 [35]. The percentage of positive cells was calculated by comparing the number of DAB-stained nuclei to the total nuclei number (hematoxylin-stained).

### Total RNA isolation

Total RNA was isolated from frozen liver (miRNeasy Mini Kit, Qiagen, UK) and quantified using a NanoDrop spectrophotometer (Thermo Scientific, USA). RNA quality and integrity were estimated by 260/280-nm and 260/230-nm ratios, showing values between 1.8–2.0 and 1.8–2.2, respectively.

### MiRNA expression

Complementary DNA (cDNA) was generated from total RNA (Taqman^TM^ MicroRNA Reverse Transcription Kit, Applied Biosystems, UK) and Taqman^TM^ MicroRNA assays (Applied Biosystems, UK). Quantitative PCR (qPCR) was performed using a Taqman^TM^ 2x Universal PCR Master Mix No AmpEraseTM UNG (Applied Biosystems, UK) on a QuantStudio 7 Flex Real-Time PCR System (Applied Biosystems, UK) with miR-582-3p (Assay ID: 472692_mat) and miR-582-5p (Assay ID: 471065_mat) probes (both Applied Biosystems, UK). MiRNA-expression assays were performed in duplicate, and data normalized to the geomean of miR-25-3p (hsa-miR-25 Assay ID: 000403, Applied Biosystems, UK), which displayed no differences between groups.

### mRNA-level assessment

Relative mRNA levels of genes encoding proteins involved in the fibrotic process, apoptosis pathway, and mitochondrial function were determined by qPCR. cDNA was synthesized using a High-Capacity cDNA Reverse Transcription Kit (Applied Biosystems, UK) and qPCR performed in duplicate using Sybr Green PCR Master Mix (Applied Biosystems, UK) on a QuantStudio 7 Flex Real-Time PCR System (Applied Biosystems, UK). Target-gene expression was normalized to the geometric mean of housekeeping genes *Ppia* and *Hprt*, as their expressions were stable between groups. Primers sequences are shown in Table 1. For measurement of both mRNA and miRNA levels, the results were expressed as the relative increase according to the comparative cycle-threshold method (2^−ΔΔCt^) [36].

**Table 1:** Primer sequences used in the qPCR.

Target genes	Forward sequence (5′-3′)	Reverse sequence (5′-3′)
*Col1a1*	GAGAGGTGAACAAGGTCCCG	AAACCTCTCTCGCCTCTTGC
*Col3a1*	TGACTGTCCCACGTAAGCAC	GAGGGCCATAGCTGAACTGA
*Col4a1*	GGCCCTTCATTAGCAGGTGT	GTGAGGACCAACCGTTAGGG
*Map3k14*	TGTCTCAAGATTGCCAGCGA	ACTTCCTGTAGTGCCTTGCC
*Tgfβ1*	CTGCTGACCCCCACTGATAC	GGGCTGATCCCGTTGATTTC
*Ndufb8*	GGTATGGCGACTACCCGATG	GTACTGCTTCGGACCCACAG
*Sdhb*	GAGTCGGCCTGCAGTTTCA	GCCAGAGTATTGCCTCCGTT
*Uqcrc2*	CCGGGTCCTTCTCGAGATTTT	TGCTTCAATCCCACGGGTTA
*Co1*	CCCAGATATAGCATTCCCACG	ACTGTTCATCCTGTTCCTGC
*Co2*	ATAACCGAGTCGTTCTGCCAAT	TTTCAGAGCATTGGCCATAGAA
*Atp5a1*	CCTTGACCTTCCTTTGCGCT	CATTTTTGGAGACCAGTCCCG
*Bax*	GAGCTGCAGAGGATGATTGC	AAGTAGAAGAGGGCAACCACG
*Bcl-2*	GCGTCAACAGGGAGATGTCA	TTCCACAAAGGCATCCCAGC
*Ampkα1*	TTCGGGAAAGTGAAGGTGGG	AGATGGTGTACTGATGACCTGG
*Ampkα2*	GGAGAACACCAATTGACAGGC	TCTTCAACCCGCCCATGTT
*Pde4d*	TGTGACATTTTCCAGAATCTGAC	AGGTTCATGTGCTTCGAC
*Nd5*	ACGAAAATGACCCAGACCTC	GAGATGACAAATCCTGCAAAGATG
*Rplp0*	AGATTCGGGATATGCTGTTGGC	TCGGGTCCTAGACCAGTGTTC
Housekeepers		
*Hprt*	GGTTAAGCAGTACAGCCCCA	GTCAAGGGCATATCCAACAACA
*Ppia*	GTCCAGGAATGGCAAGACCA	GGGTAAAATGCCCGCAAGTC

### Protein extraction and Western blotting

Hepatic expression of proteins implicated in fibrosis, apoptosis, mitochondrial function, and oxidative stress was assessed by western blotting [20]. Each membrane was blotted with the respective primary antibody [p-SAPK/JNK (Thr183/Tyr185) (#9251, Cell Signaling Technology, USA), Bax (#2772, Cell Signaling Technology, USA), Bcl-2 (#2876, Cell Signaling Technology, MA, USA), Total OXPHOS (ab110413, Abcam, UK), Citrate Synthase (CS; Cat. 16131-1-AP, Proteintech, UK), Catalase (ab1877, Abcam, UK), MnSOD (06-984, Merck Millipore, UK), *p*-AMPKα (Thr172) (#2535, Cell Signaling Technology, USA), and AMPKα1 (ab3759, Abcam, UK)]. Proteins were detected with horseradish peroxidase-conjugated anti-rabbit or anti-mouse secondary antibody (Jackson Immuno Research, UK). Detection was performed with Super Signal West Pico Chemiluminescent substrate (Thermo Scientific, UK) using an ImageQuant^TM^ LAS 4000 (GE Healthcare Life Sciences, UK). Band intensities were quantified by optical densitometry (Scion Image-Release Beta 3b, NIH, USA). To confirm equal protein loading, membranes were stripped (Restore™ Western Blot Stripping Buffer, Thermo Scientific, UK) and reblotted with an anti-GAPDH antibody (#2118, Cell Signaling Technology, USA). Western blotting data were calculated relative to the CC group, which were defined as 100 percent.

### Relative mitochondrial DNA content

Copy number of mitochondrial DNA is one biomarker of mitochondrial dysfunction [37]. Total DNA was extracted from liver (DNeasy Blood & Tissue kit, Qiagen, UK) and total double-stranded DNA concentrations determined (Quant-iT^TM^ PicoGreen dsDNA assay kit, Invitrogen, UK). Mitochondrial DNA content was determined by the ratio between a mitochondrial (*Nd5*) and nuclear (*Rplp0)* gene following qPCR. Primer sequences are in Table 1. The results were expressed using the following equations: ΔC_T_ = (nuclear DNA C_T_ - mitochondrial DNA C_T_) and relative mitochondrial DNA content = 2 *x* 2 ΔC_T_ [38].

### Determination of mitochondrial respiratory chain (MRC) enzyme activities

Hepatic MRC enzyme activities were evaluated spectrophotometrically as described previously: complex I [39], complex II/III [40], and complex IV [41]. Complex activities were expressed as a ratio to CS [42] to correct for mitochondrial enrichment of the samples [43].

### Malondialdehyde (MDA) concentration

Malondialdehyde, a marker for lipid peroxidation, was quantified fluorometrically (Ex/Em=532/553 nm) (ab118970, Abcam, UK) and normalized to protein content of the sample.

### Statistical analysis

Power calculations for determining adequate sample size were based on published results from our group [32], and in order to detect a 20% difference in means with a power (95% confidence and alpha = 0.05) of 0.9, the sample size required per group was determined to be *n* = 9 dams per group (mother is the statistical unit). Shapiro–Wilk quality test was used to assess the normality of the data. Grubbs’ test was performed and significant outlier samples were removed when the alpha value is equal to 0.05 [44]. Normally distributed data were analyzed using an unpaired two-tailed student’s *t* test. Data with nonnormal distribution were analyzed by the two-tailed Mann–Whitney U test or by the two-tailed Kolmogorov–Smirnov test. Two-way analysis of variance (ANOVA) was performed to estimate the effect of two independent variables (maternal diet and offspring age) followed by Bonferroni post hoc test. A chi-square one-sided test was used to analyze the binary data generated from an assessment of fibrosis by a pathologist. Pearson (normal distribution) or Spearman (nonnormal distribution) correlation coefficients were also determined. All statistical analyses were performed using GraphPad Prism 7.02 for Windows (GraphPad Software, USA). Data are presented as mean ± standard error of the mean (SEM) and differences considered significant when *p* < 0.05.

## Results

### Body weight and composition

**Fig. 1:**
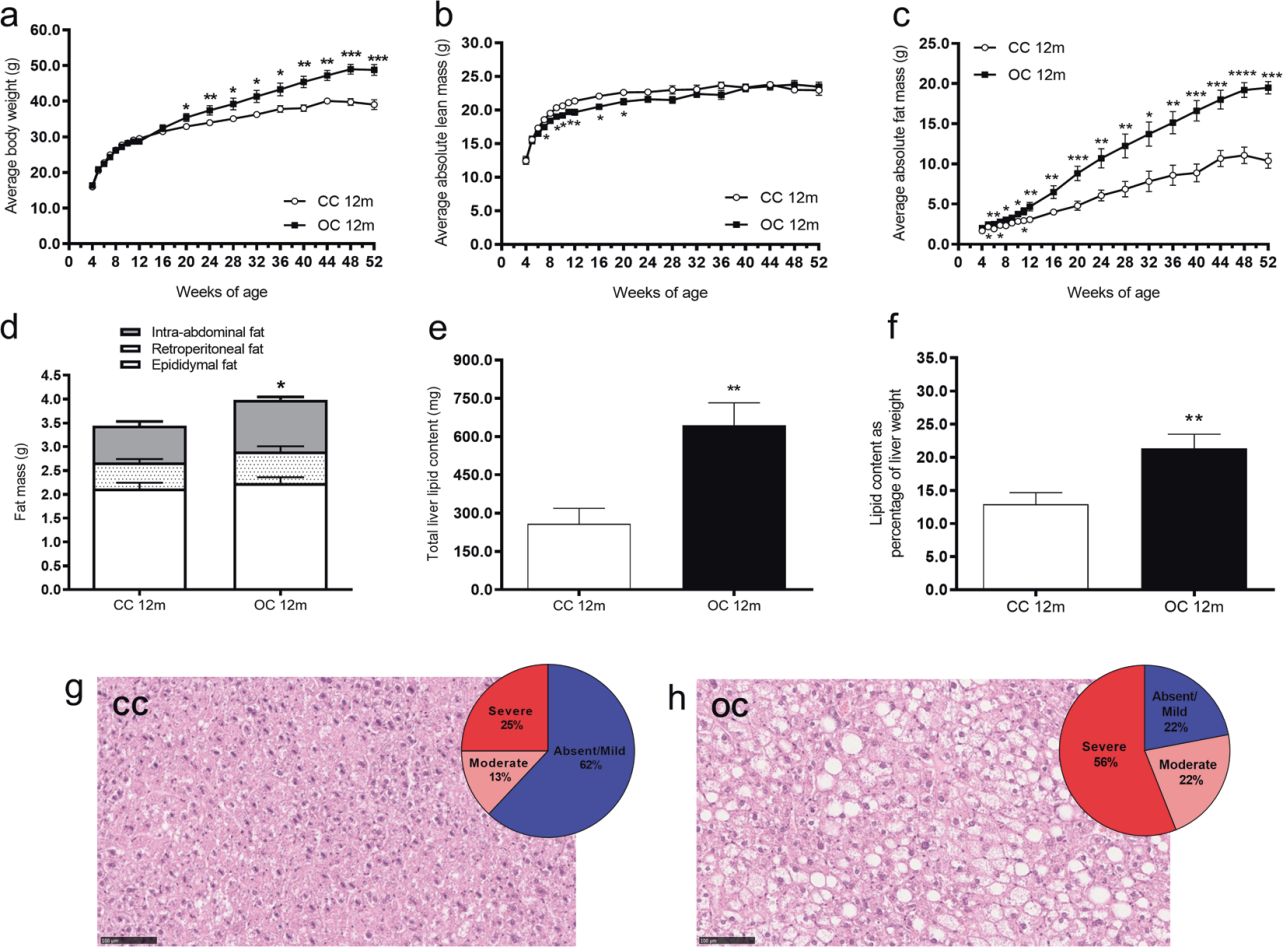
Body weight, body composition, hepatic lipid content and steatosis grades in 12-month-old offspring. Average body weight (**a**), average absolute lean mass (**b**), average absolute fat mass (**c**), fat mass of intra-abdominal, retroperitoneal and epididymal fat depots (**d**), total hepatic lipid content (**e**), hepatic lipid content as percentage of liver weight (**f**), representative liver sections stained with H&E and percentage of steatosis grades in the CC group (**g**) and OC group (**h**) at 12 months of age. CC 12 m: mother received control diet and offspring-fed control diet after weaning up to 52 weeks of age; OC 12 m: mother received obesogenic diet and offspring-fed control diet after weaning up to 52 weeks of age. Values are presented as means ± SEM; CC 12 m: *n* = 7–13 and OC 12 m: *n* = 8–9 animals from independent litters for each group. Data were analyzed by unpaired student’s *t* test or Mann–Whitney U test. *p < 0.05, **p < 0.01, ***p < 0.001, and ****p < 0.0001.

In both groups, body weight and body-fat content increased with age (effect of age: *p* < 0.0001), however, the effect was greater in the offspring of obese dams (interaction between maternal diet and age: *p* < 0.0001) (Fig. 1a). Therefore, at 12 months of age, offspring of obese dams were significantly heavier (CC 39.1 ± 1.4 g versus OC 48.8 ± 1.5 g, *p* < 0.0001; CC: *n* = 9 and OC: *n* = 8) and fatter (CC 10.4 ± 0.9 g versus OC 19.5 ± 0.8 g, *p* < 0.0001; CC: *n* = 9 and OC: *n* = 8) than control offspring. There was a small reduction in the absolute lean mass from week 7 to week 20 in the OC group; however, from 24 weeks of age until the end of experimental period, the lean mass was similar between groups (Fig. 1b). The difference in fat mass between the groups became more marked as the animals aged (Fig. 1c). There was a depot-specific difference in fat deposits, with the greatest difference observed in the intra-abdominal depot of the OC group at 12 months of age (Fig. 1d).

### Fasting-serum metabolites, HOMA-IR and GTT (AUC) in 12-month-old offspring

The level of circulating TG was significantly lower in the OC group compared with the CC group (Table 2). Additionally, offspring from obese dams exhibited increased serum concentrations of LDL and leptin. There were no significant differences between offspring groups for total cholesterol, HDL, FFA, insulin, fasting glucose, HOMA-IR, or glucose tolerance (Table 2).

**Table 2:** Fasting serum metabolites, HOMA-IR and GTT (AUC) in the offspring at 12 months of age.

	CC 12 m	OC 12 m	*p* value
Triglycerides (mmol L^−1^)	1.38 ± 0.13	1.06 ± 0.07*	0.0448
Total cholesterol (mmol L^−1^)	3.20 ± 0.26	4.12 ± 0.3 1	0.0720
HDL (mmol L^−1^)	1.65 ± 0.13	1.93 ± 0.11	0.2224
LDL (mmol L^−1^)	0.92 ± 0.15	1.71 ± 0.23*	0.0119
Free fatty acids (µmol L^−1^)	953 ± 87	798 ± 48	0.1366
Insulin (pmol L^−1^)	380 ± 55	507 ± 85	0.2266
Leptin (pmol L^−1^)	4097 ± 332	7181 ± 723**	0.0017
Fasting glucose (mmol L^−1^)	9.4 ± 0.6	10.0 ± 0.8	0.5153
HOMA-IR	0.13 ± 0.02	0.19 ± 0.03	0.1425
GTT (AUC)	29.7 ± 2.5	29.9 ± 0.8	0.9510

AUC Area Under the Curve, HDL high-density lipoprotein, HOMA-IR Homeostatic Model Assessment for Insulin Resistance, LDL low-density lipoprotein, GTT Glucose Tolerance Test. CC 12 m: mother received control diet and offspring fed control diet after weaning up to 52 weeks of age; OC 12 m: mother received obesogenic diet and offspring fed control diet after weaning up to 52 weeks of age. Values are presented as means ± SEM. CC 12 m: *n* = 8–9 and OC 12 m: *n* = 7–9 from independent litters for each group. Data were analyzed by unpaired student’s *t* test or Mann–Whitney U test. *p < 0.05; **p < 0.01*.*

### Liver weight, hepatic lipid content, and steatosis grade in 12-month-old offspring

Absolute liver weight was increased in OC offspring compared with CC offspring at 12 months of age (CC 1.8 ± 0.2 g versus OC 2.9 ± 0.3 g, *p* < 0.01; CC: *n* = 9 and OC: *n* = 9). The increase remained when liver weight was presented as percentage of body weight (CC 4.36 ± 0.22% versus OC 6.36 ± 0.28%, *p* < 0.0001; CC: *n* = 9 and OC: *n* = 8). The offspring of obese dams showed elevated total hepatic lipid content at 12 months of age (Fig. 1e). Increased hepatic lipid content was also apparent when the results were expressed as percentage of liver weight (Fig. 1f). Blinded histopathological assessment revealed that 56% of offspring from obese dams developed severe steatosis at 12 months of age, while only 25% of control offspring had this degree of steatosis. Likewise, the percentages of animals with moderate steatosis were also greater in the OC group (22% versus 13%). Over half (62%) of control animals (CC) had absent/mild steatosis, whereas only 22% offspring of obese (OC) dams had absent/mild steatosis (Figs. 1g and 1h).

### AMP-activated protein-kinase α (AMPKα) expression in the liver of 12-month-old offspring

**Fig. 2:**
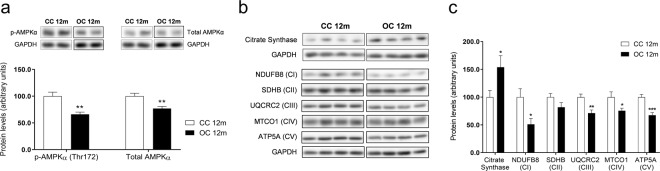
AMPKα protein expression and mitochondrial phenotype in the liver of 12-month-old offspring. Protein levels of p-AMPKα (Thr172) and total AMPKα (**a**), representative images of protein expression (**b**), and relative protein expression of citrate synthase and mitochondrial OXPHOS complexes (**c**) in the liver of the offspring at 12 months of age. CC 12 m: mother received control diet and offspring-fed control diet after weaning up to 52 weeks of age; OC 12 m: mother received obesogenic diet and offspring-fed control diet after weaning up to 52 weeks of age. Values are presented as means ± SEM; CC 12 m: *n* = 9 and OC 12 m: *n* = 9 animals from independent litters for each group. Data were analyzed by unpaired student’s *t* test. *p < 0.05, **p < 0.01, and ***p < 0.001.

We observed significant reductions in both phosphorylation (Thr172) and total protein levels of AMPKα in the OC group (Fig. 2a). The differences in protein expression were not accompanied by any differences in relative mRNA levels of *Ampkα1* (CC 1.00 ± 0.04 versus OC 1.00 ± 0.06; CC: *n* = 9 and OC: *n* = 9) or *Ampkα2* (CC 1.00 ± 0.04 versus OC 1.10 ± 0.05; CC: *n* = 9 and OC: *n* = 9). Additionally, liver lipid content strongly negatively correlated with p-AMPKα (*r* = −0.7375, *p* = 0.0005), total AMPKα (*r* = −0.5974, *p* = 0.0088), and AMPKα activity (*r* = −0.6697, *p* = 0.0033) (Supplementary Fig. 1a–c, respectively).

### Mitochondrial phenotype in the liver of 12-month-old offspring

There was a significant reduction in protein expression of OXPHOS MRC complexes I (NDUFB8), III (UQCRC2), IV (MTCO1), and V (ATP5A) and an increase in hepatic protein levels of citrate synthase in the OC group (Figs. 2b and 2c). In contrast, the OC group had increased mRNA levels of *Uqcrc2* (complex III: CC 1.00 ± 0.03 versus OC 1.12 ± 0.05, *p* < 0.05, CC: *n* = 9 and OC: *n* = 9) and *Atp5a1* (complex V: CC 1.00 ± 0.04 versus OC 1.12 ± 0.04, *p* < 0.05; CC: *n* = 9 and OC: *n* = 9). mRNA expression of *Ndufb8* (complex I: CC 1.00 ± 0.02 versus OC 1.01 ± 0.03, CC: *n* = 8 and OC: *n* = 9), *Sdhb* (complex II: CC 0.93 ± 0.04 versus OC 1.00 ± 0.04, CC: *n* = 9 and OC: *n* = 9), and *Co1* (complex IV: CC 1.01 ± 0.08 versus OC 1.32 ± 0.20; CC: *n* = 9 and OC: *n* = 9) and *Co2* (complex IV: CC 1.06 ± 0.13 versus OC 1.00 ± 0.12, CC: *n* = 9 and OC: *n* = 9) was not different between the groups. There was no change in the relative mitochondrial DNA content (CC 42.7 ± 2.7 versus OC 41.1 ± 2.0, CC: *n* = 9 and OC: *n* = 9) or in the activity of MRC complexes (complex I: CC 0.87 ± 0.05 versus OC 0.88 ± 0.05 relative to CS activity; CC: *n* = 9 and OC: *n* = 9; complexes II–III: CC 0.12 ± 0.01 versus OC 0.15 ± 0.02 relative to CS activity, CC: *n* = 9 and OC: *n* = 9; complex IV: CC 0.08 ± 0.01 versus OC 0.08 ± 0.01 k/nmol, CC: *n* = 9 and OC: *n* = 9) between groups.

We also examined hepatic lipid peroxidation and oxidative enzyme. However, there were no differences in MDA concentration (CC 0.20 ± 0.01 versus OC 0.22 ± 0.01 nmol/mg of protein, CC: *n* = 9 and OC: *n* = 9) or in protein levels of catalase (CC 100 ± 12 versus OC 92 ± 11, CC: *n* = 9 and OC: *n* = 9) or MnSOD (CC 100 ± 9 versus OC 99 ± 13, CC: *n* = 8 and OC: *n* = 9) between groups.

### Hepatic fibrosis and apoptosis in 12-month-old offspring

**Fig. 3:**
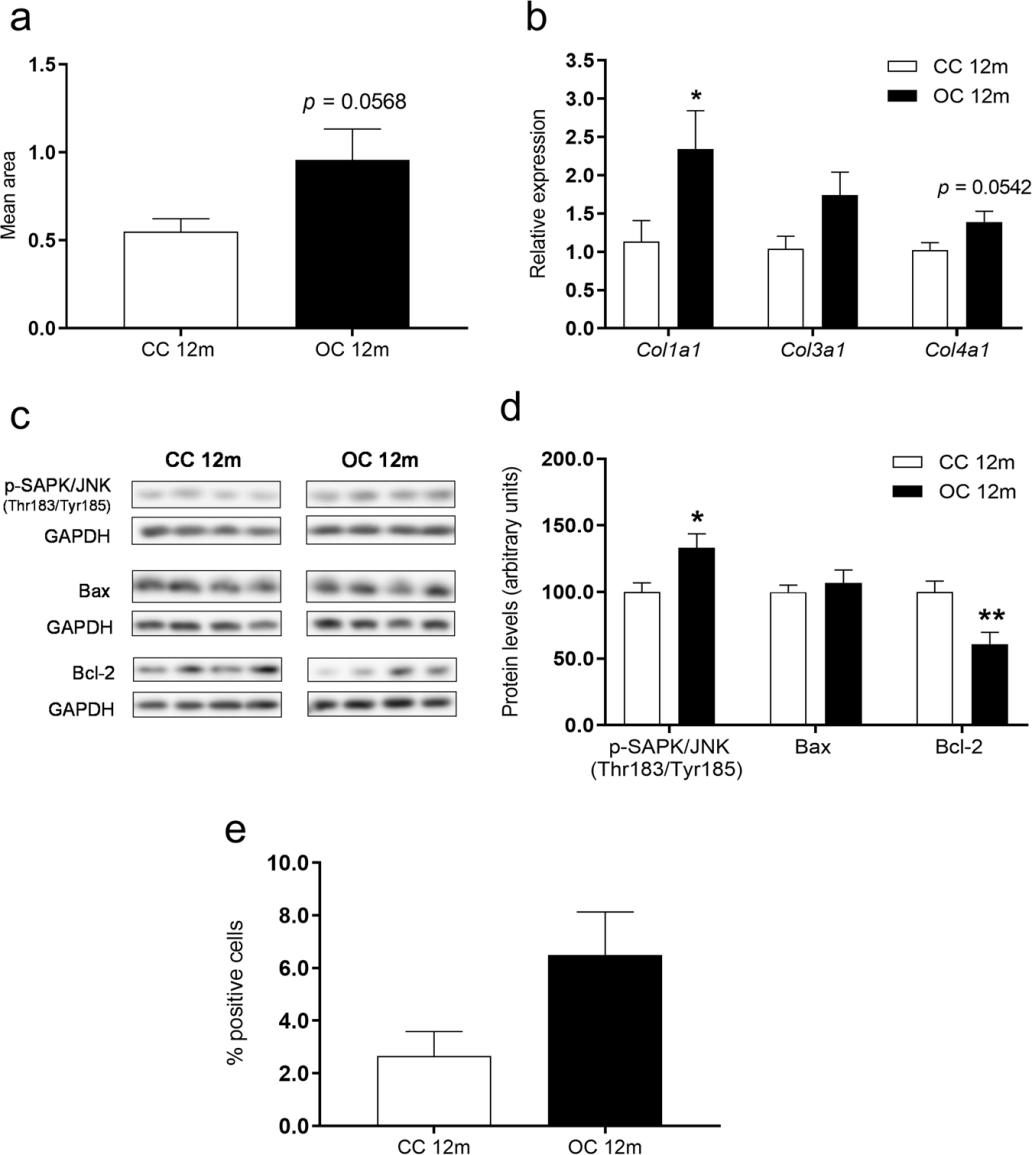
Hepatic fibrosis and apoptosis in 12-month-old offspring. Quantification of Picrosirius red staining (**a**), relative mRNA levels of *Col1a1, Col3a1* and *Col4a1* (**b**), representative images of protein expression (**c**), relative protein expression of p-SAPK/JNK, Bax and Bcl-2 (**d**), and percentage of TUNEL-positive cells (**e**) in the liver of the offspring at 12 months of age. CC 12 m: mother received control diet and offspring-fed control diet after weaning up to 52 weeks of age; OC 12 m: mother received obesogenic diet and offspring fed control diet after weaning up to 52 weeks of age. Values are presented as means ± SEM; CC 12 m: *n* = 7–9 and OC 12 m: *n* = 8–9 animals from independent litters for each group. Data were analyzed by unpaired student’s *t* test or Mann–Whitney U test or Kolmogorov–Smirnov test. *p < 0.05 and **p < 0.01.

To investigate the development of fibrosis in offspring liver at 12 months of age, quantification of collagen accumulation (Supplementary Fig. 2a and 2b) and a blinded histopathologic evaluation of fibrosis were carried out. We did not observe extensive collagen staining in either experimental group, but OC offspring exhibited a borderline significant (*p* = 0.0568) increase in hepatic collagen deposition (Fig. 3a). Consistent with low levels of fibrosis, histological scoring revealed no moderate or severe fibrosis in any livers of either group. A centrilobular/pericellular fibrosis (classified formally as mild) was detected in 3/9 animals from the OC group, while all control samples were deemed as fibrosis-absent (*p* < 0.05, CC: *n* = 7 and OC: *n* = 9). Relative mRNA levels of *Col1a1* were significantly (*p* < 0.05) increased and *Col4a1* was borderline significantly increased (*p* = 0.0542) in the obese offspring (Fig. 3b). Despite displaying a similar pattern, *Col3a1* gene expression was not significantly changed between experimental groups (Fig. 3b). There were increased levels of phosphorylated c-Jun-N-terminal kinase (p-SAPK/JNK) (Figs. 3c and 3d), as well as relative mRNA levels of transforming growth factor-β (*Tgfβ1*) (CC 1.01 ± 0.07 versus OC 1.26 ± 0.09, *p* < 0.05; CC: *n* = 9 and OC: *n* = 9) and NF-κB-inducing kinase (*Map3k14*) (CC 1.02 ± 0.08 versus OC 1.38 ± 0.09, *p* < 0.01; CC: *n* = 9 and OC: *n* = 9) in the OC group. We also investigated some key mediators of the apoptotic pathway. Offspring from obese dams showed decreased protein expression of Bcl-2 (Figs. 3c and 3d) and an increased Bax/Bcl-2 ratio (CC 1.02 ± 0.07 versus OC 1.64 ± 0.24, *p* < 0.05; CC: *n* = 9 and OC: *n* = 8). Concomitantly, there was a negative correlation between Bcl-2 protein levels and hepatic collagen deposition (*r* = −0.6436, *p* = 0.0053) (Supplementary Fig. 1d). Protein levels of Bax (Figs. 3c and 3d) and transcript levels of both *Bax* (CC 1.00 ± 0.03 versus OC 1.08 ± 0.03; CC: *n* = 9 and OC: *n* = 9) and *Bcl-2* (CC 1.03 ± 0.09 versus OC 1.18 ± 0.12; CC: *n* = 9 and OC: *n* = 9) were not significantly different between CC and OC groups. The percentage of TUNEL-positive cells was low in both groups and not significantly different (*p* = 0.0807) (Fig. 3e).

### MiR-582 expression in the liver of 12-month-old offspring

**Fig. 4:**
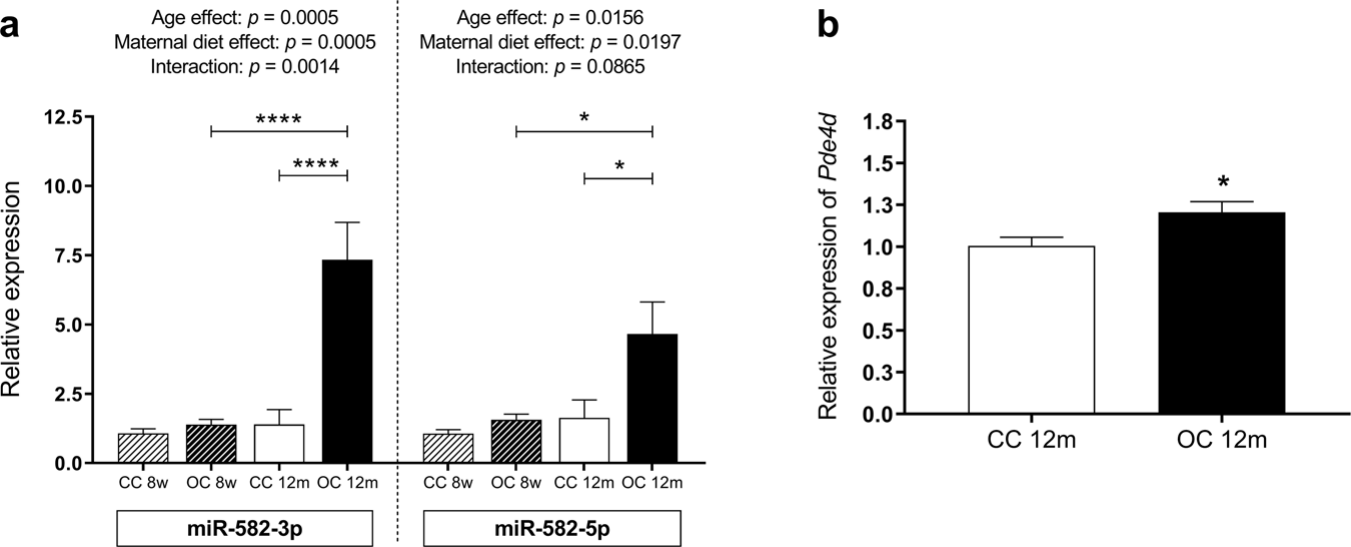
Age-related changes in the miR-582 hepatic expression in the 8-week-old and 12-month-old offspring. Relative expressions of miR-582-3p and miR-582-5p in the liver of the offspring at eight weeks and 12 months of age (**a**) and relative mRNA expression of phosphodiesterase 4D in the liver of the offspring at 12 months of age (**b**). CC 8w: mother received control diet and offspring-fed control diet after weaning up to eight weeks of age; OC 8w: mother received obesogenic diet and offspring-fed control diet after weaning up to eight weeks of age. CC 12 m: mother received control diet and offspring-fed control diet after weaning up to 52 weeks of age; OC 12 m: mother received obesogenic diet and offspring-fed control diet after weaning up to 52 weeks of age. Values are presented as means ± SEM; CC 8w: *n* = 8 and OC 8w: *n* = 8; CC 12 m: *n* = 8–9 and OC 12 m: *n* = 9 animals from independent litters for each group. Data were analyzed by Mann–Whitney U test or two-way ANOVA. *p < 0.05 and ****p < 0.0001.

Maternal diet-induced obesity during pregnancy and lactation resulted in increased expression of both miR-582-3p and miR-582-5p in the older-programmed offspring (Fig. 4a). There was also a significant effect of age to increase both miR-582-3p and miR-582-5p. However, in both cases, the impact of age was greater in the offspring of obese dams compared with controls, meaning that the greatest effect of maternal diet was at 12 months of age when hepatic expression of miR-582-3p and miR-582-5p was substantially higher in the offspring of the obese dams (Fig. 4a). MiR-582 is intragenic, located within the gene phosphodiesterase 4D (*Pde4d*). Similarly, to the miRNA expression, expression of *Pde4d* was increased in the 12-month-old offspring from obese dams (Fig. 4b). However, the magnitude of increase in the host mRNA was much smaller than that of the miRNAs. Both strands of mmu-miR-582 (-3p and -5p) were positively correlated with hepatic lipid accumulation (*r* = 0.7549, *p* = 0.0007 and *r* = 0.7420, *p* = 0.0004, respectively) (Supplementary Fig. 1e and 1f).

## Discussion

Our study revealed that maternal diet-induced obesity caused by consumption of a high-fat/high-sugar diet adversely programs growth trajectory, adiposity, hepatic lipid accumulation, and moderate/severe steatosis in 12-month-old offspring fed a control diet from weaning. Our experimental design does not allow for the differentiation of effects resulting from maternal obesity per se and those caused by maternal consumption of a high-fat/high-sugar diet. However, in the human situation, these often occur in parallel. Our findings highlight the importance of advancing age in the development of programmed phenotype, with longitudinal data demonstrating that the effects on body weight and body composition become more pronounced with age. Consistent with this observation, there is an interaction between maternal diet and age on expression of miR-582 with the effect of age being exaggerated in the male offspring exposed to maternal obesity. These observations highlight the importance of studying the impact of programming across the life course.

Previous studies from our research group using the same model demonstrated that chow-fed offspring have similar body weights up to 12 weeks of age [22, 45–47]. In the current study, we observed a significant impact of age, with the offspring of obese dams displaying increased body weight and fat mass from around 20 weeks of age and that the magnitude of effect increased up to 12 months of age. Consistent with these findings, Rodríguez-González et al. [25] demonstrated that postnatal day 450 was the first age at which body weight and adiposity index were greater in male-rat offspring of a similar maternal obesity model. In addition to the effects on adiposity, the current study also showed that programmed changes in metabolites were associated with advancing age. Previous studies on 8-week-old mice showed no effects of maternal obesogenic diets on serum LDL cholesterol and leptin levels in offspring of obese dams [47]. However, both were elevated as a consequence of maternal obesity at 12 months of age. Persistent elevation in circulating LDL increases the risk of CVD development and LDL-particle concentration has been positively associated with NAFLD [48, 49]. Therefore, high LDL concentration observed in the older offspring may contribute to programming of cardiovascular and hepatic diseases.

NAFLD refers to excessive intrahepatocellular fat deposition and is considered to be the liver manifestation of metabolic syndrome [50]. NAFLD is one of the most prevalent liver diseases in the world and evidence suggests that an adverse early life environment can program offspring susceptibility to the development and progression of this condition [9]. In the current study, increased hepatic lipid content, liver weight, and percentage of animals with severe-to-moderate steatosis (78% in total) were accompanied by increased body weight and adiposity in the older progeny exposed to a maternal obesogenic environment, indicating the presence of NAFLD independently of changes in the fasting concentrations of FFA, total cholesterol, HDL, insulin, or glucose tolerance. Despite increased hepatic lipid accumulation, offspring of obese dams had reduced serum levels of circulating triglycerides. The underlying mechanisms are unknown but may indicate an inability to export triglycerides from the liver into the circulation or an enhanced uptake of triglycerides by adipose tissue. Similar observations have been made in other mouse models of nonalcoholic steatohepatitis, such as those using a choline-deficient L-amino acid-defined high-fat diet [51].

Given that strong negative correlations were found between hepatic p-AMPKα protein levels and liver lipid content, as well as between the p-AMPKα/AMPKα ratio and liver lipid content, we speculate that the hepatic fat accumulation observed in these offspring can be partially explained by defects in AMPKα activation and activity. It has been shown previously that AMPK activation declines with obesity [52] and aging [53], which is consistent with the offspring of obese dams displaying an obese, prematurely metabolic aging phenotype. Furthermore, it has been reported that exogenous AMPK activation can be a viable strategy for the treatment of obesity and NAFLD [54]. In the current study, the absence of differences in the relative mRNA levels of *Ampkα1* or *Ampkα2* may suggest a post-transcriptional mechanism mediating the expression of AMPK.

Mitochondria dysfunction has been implicated in developmental programming and age-related diseases [55–57]. Previous studies showed decreased protein levels of mitochondrial respiratory chain complex II, complex III, and complex V in the liver [57] and skeletal muscle [55] of young offspring (at weaning [57] and eight weeks of age [55]) born to obese dams. In the current study, we demonstrated reductions in a number of proteins within these complexes; however, there were no differences in mitochondrial copy number or enzyme activity. Protein subunits NDUFB8 (complex I) [58] and UQCRC2 (complex III) [59] are structural subunits without involvement in the catalytic activity. Therefore, although there was a significant decrease in protein levels of NDUFB8 and UQCRC2, it may not impact on the MRC enzyme activity but impacts on other aspects of mitochondrial biology. Our findings also revealed that increased mRNA levels of *Uqcrc2* and *Atp5a1* may indicate a compensatory response to the reduction in the protein expression of these MRC complexes in the obese offspring. In parallel, the absence of significant changes in the mRNA levels of *Ndufb8, Co1*, and *Co2* again suggests that differences in protein expression may arise from post-transcriptional mechanisms.

The spectrum of NAFLD in humans ranges from nonalcoholic fatty liver (NAFL, simple hepatic steatosis) to NASH (severe form of the disease). NAFL can progress to NASH, which is characterized by hepatocellular injury and inflammation with or without fibrosis [50]. Liver fibrosis results from an excessive accumulation of collagen and other extracellular-matrix proteins in the affected tissue [60]. Mice models generally do not develop NASH and moderate or severe fibrosis is rarely reported [61], consistent with the absence of moderate/severe fibrosis in the current study. However, we demonstrated increased collagen deposition, gene overexpression of *Col1a1*, and mild centrilobular/pericellular fibrosis in some animals, indicating that maternal diet-induced obesity leads to increased risk of liver fibrosis in the older offspring at 12 months of age, which could progress if the animals were aged further. Leptin, obesity, and TGFβ play a crucial role in the profibrogenic responses within the liver [60, 62, 63]. Leptin is thought to upregulate the expression of TGFβ, which in turn activates JNK, leading to fibrosis and apoptosis [62, 63]. Consistent with this, we revealed increased circulating leptin and increased hepatic TGFβ as well as increased phosphorylated JNK.

Apoptosis is considered a key mediator of NAFLD progression and the degree of apoptosis is inversely associated with the level of Bcl-2 [64, 65]. The Bcl-2 family includes three groups: anti-apoptotic proteins (Bcl-2), pro-apoptotic pore formers (Bax) and pro-apoptotic BH3-only proteins [66]. The balance between anti- and pro-apoptotic members is crucial [66] and a high Bax/Bcl-2 ratio, as observed in the livers from the offspring of obese dams, suggests a pro-apoptotic phenotype [67]. Although we detected decreased protein level of Bcl-2 (an important apoptosis-regulatory protein) and high Bax/Bcl-2 ratio in the animals from OC group, the percentage of TUNEL-positive cells was low and did not differ between groups at 12 months of age. These findings may suggest that hepatic cells from obese offspring are more susceptible to apoptotic stimuli; however, the time point investigated was too early to detect differences in the number of apoptotic cells by TUNEL assay. Furthermore, the strong negative correlation between Bcl-2 protein expression and collagen deposition in the liver supports an interaction between liver fibrosis and apoptosis.

We demonstrated that maternal diet-induced obesity leads to differences in hepatic expression of miR-582 (-3p and -5p) in the 12-month-old offspring of obese dams. This miRNA has not previously been implicated in the programming of liver, but has been shown to be increased in response to high-fructose-diet consumption [17] and in cirrhotic livers [18]. It has also been shown to be increased in the cardiac left ventricle of gestational protein-restricted offspring at 12 days of age [68] and in maternal plasma from women with preeclampsia [69]. In the current study, we demonstrated an impact of advancing age on hepatic miR-582-3p and miR-582-5p with levels increasing between offspring at eight weeks (young adult just after sexual maturity) and 12 months (older adults at middle age). However, the magnitude of the increase was much more marked in the offspring of obese dams compared with control offspring. This indicates that disturbances in the expression of these miRNAs are also consistent with an accelerated metabolic aging process triggered by a maternal obesogenic environment prior to and during pregnancy and lactation.

There are some limitations to acknowledge in the current study. First, there is growing evidence that male and female fetuses respond differently to a suboptimal in utero environment, leading to sexually dimorphic programmed responses [70]. In the current study, only males were included and, therefore, it remains unknown if the changes observed would also occur in female offspring. Second, although studying mice at an older age (12 months) than most other programming studies, this still represents approximately half of the mouse lifespan. Therefore, further studies are required to establish if age-associated changes become even great as the animals near their expected lifespan.

In conclusion, we demonstrate that maternal diet-induced obesity unfavorably modulates body composition and fatty-liver phenotype in exposed offspring that is exaggerated with age. This is accompanied by an accelerated age-associated increase in hepatic levels of miR-582, which may contribute mechanistically to the development of hepatic fatty liver. Our findings support the hypothesis that accelerated metabolic aging may contribute to the development of programmed phenotypes and highlights the possibility that there may be a critical time for intervention after the suboptimal exposure and prior to the development of programmed metabolic dysfunction.

## Supplementary information

Supplementary material figure legends

Supplementary material (Figure 1)

Supplementary material (Figure 2)
