# Supplementary material for: Effects of maternal diet-induced obesity on metabolic disorders and age-associated miRNA expression in the liver of male mouse offspring
Source: Int J Obes (Lond). 2021 Oct 18;46(2):269–78. doi: 10.1038/s41366-021-00985-1 (PMC8794789; doi:10.1038/s41366-021-00985-1)
Supplement: Supplementary file 1 — Supplementary material figure legends [file 41366_2021_985_MOESM1_ESM.docx]

**Supplementary material figure legends**

**Supplementary Fig. 1** Pearson correlations plots (a-d) and Spearman correlations plots (e and f).

**Supplementary Fig. 2** Representative liver sections stained with Picrosirius Red in the CC group (a) and OC group (b) at 12 months of age. Images presented were 20 times magnification. Scale bars represent 100 μm.
