# Supplementary figures and images for: Effects of maternal diet-induced obesity on metabolic disorders and age-associated miRNA expression in the liver of male mouse offspring
Source: Int J Obes (Lond). 2021 Oct 18;46(2):269–78. doi: 10.1038/s41366-021-00985-1 (PMC8794789; doi:10.1038/s41366-021-00985-1)

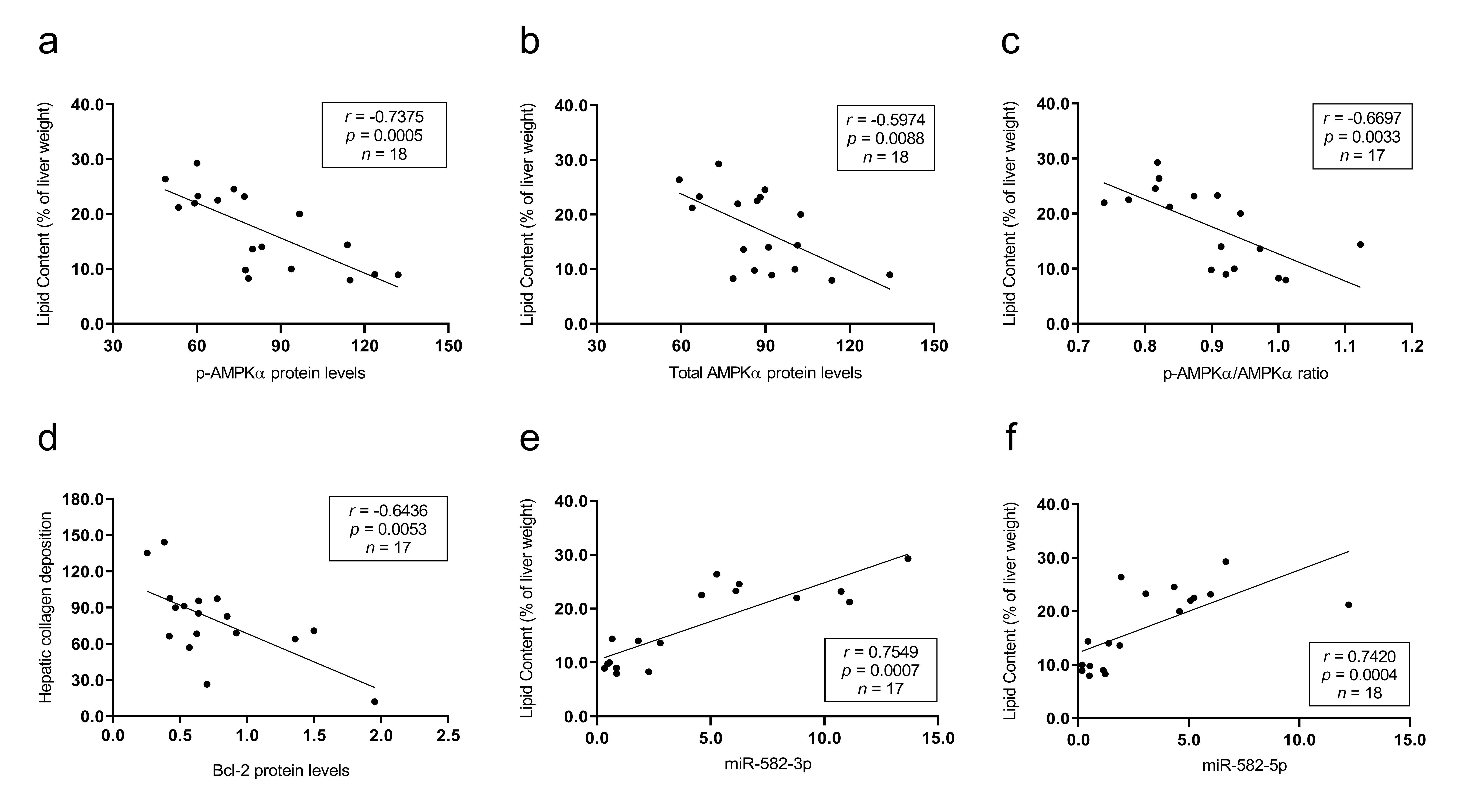

Supplement: Supplementary file 2 — Supplementary material (Figure 1) [file 41366_2021_985_MOESM2_ESM.tif]

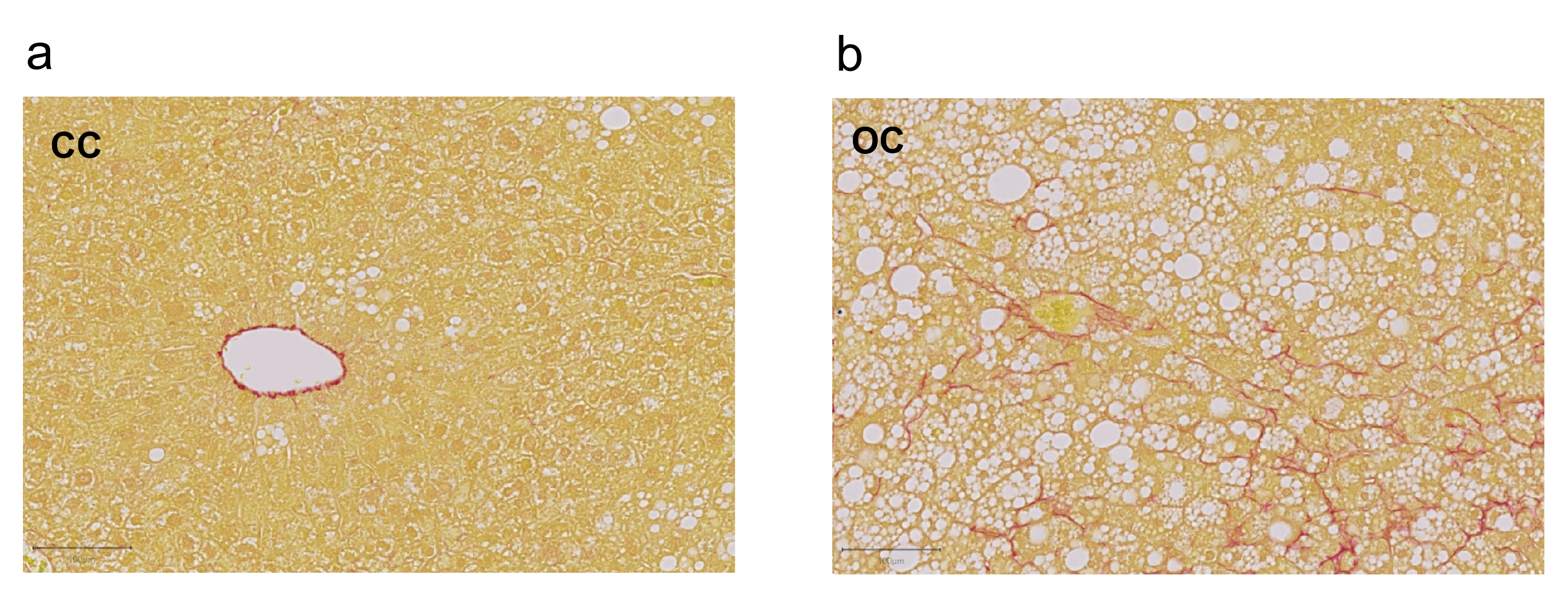

Supplement: Supplementary file 3 — Supplementary material (Figure 2) [file 41366_2021_985_MOESM3_ESM.tif]
